# SeedMatchR: identify off-target effects mediated by siRNA seed regions in RNA-seq experiments

**DOI:** 10.1093/bioinformatics/btae011

**Published:** 2024-01-08

**Authors:** Tareian Cazares, Richard E Higgs, Jibo Wang, Hatice Gulcin Ozer

**Affiliations:** Genetic Medicine, Eli Lilly and Company, Indianapolis, IN 46225, United States; Genetic Medicine, Eli Lilly and Company, Indianapolis, IN 46225, United States; Genetic Medicine, Eli Lilly and Company, Indianapolis, IN 46225, United States; Genetic Medicine, Eli Lilly and Company, Indianapolis, IN 46225, United States

## Abstract

**Motivation:**

On-target gene knockdown, using siRNA, ideally results from binding fully complementary regions in mRNA transcripts to induce direct cleavage. Off-target siRNA gene knockdown can occur through several modes, one being a seed-mediated mechanism mimicking miRNA gene regulation. Seed-mediated off-target effects occur when the ∼8 nucleotides at the 5’ end of the guide strand, called a seed region, bind the 3’ untranslated regions of mRNA, causing reduced translation. Experiments using siRNA knockdown paired with RNA-seq can be used to detect siRNA sequences with off-target effects driven by the seed region. However, there are limited computational tools designed specifically for detecting siRNA off-target effects mediated by the seed region in differential gene expression experiments.

**Results:**

*SeedMatchR* is an R package developed to provide users a single, unified resource for detecting and visualizing seed-mediated off-target effects of siRNA using RNA-seq experiments. *SeedMatchR* is designed to extend current differential expression analysis tools, such as *DESeq2*, by annotating results with predicted seed matches. Using publicly available data, we demonstrate the ability of *SeedMatchR* to detect cumulative changes in differential gene expression attributed to siRNA seed region activity.

**Availability:**

*SeedMatchR* is available on CRAN. Documentation and example workflows are available through the *SeedMatchR* GitHub page at https://github.com/tacazares/SeedMatchR.

## 1 Introduction

RNA interference approaches utilizing small interfering RNA (siRNA) to downregulate gene expression have shown efficacy for the treatment of genomic-related diseases (Hu *et al.* 2020, Alshaer *et al.* 2021, de Brito *et al.* 2022, Schlegel *et al.* 2022). Argonaut (AGO) proteins complex with siRNA to form the machinery that targets transcripts with full sequence complementarity to the siRNA antisense sequence (guide) for degradation (Gorski *et al.* 2017, Alshaer *et al.* 2021, Kobayashi *et al.* 2021, Kobayashi *et al.* 2022). Knockdown of mRNA due to full sequence complementarity is considered an on-target hit (Alshaer *et al.* 2021, Kobayashi *et al.* 2021, 2022, Schlegel *et al.* 2022). MicroRNA (miRNA) are small non-coding RNA that also control gene expression through the AGO system by binding with varying degrees of sequence complementarity to the 3’ UTR regions of protein coding transcripts (Bartel 2009, Gorski *et al.* 2017, McGeary *et al.* 2019). The 5’ end of the guide sequence of both miRNA and siRNA, called a seed region, is responsible for mediating site recognition (Bartel 2009, Kobayashi *et al.* 2022). The seed region is roughly defined as the first eight nucleotides of the 5’ end of the guide sequence (Bartel 2009). Binding of the seed region to the 3’ UTR region of transcripts can result in repression of protein translation, as opposed to the cleavage-mediated degradation of transcripts by siRNA complementarity (McGeary *et al.* 2019, Alshaer *et al.* 2021, Kobayashi *et al.* 2021, 2022). Due to overlapping mechanisms of action between siRNA and miRNA guide sequences, siRNA can also take on a miRNA like mechanisms of gene repression that are considered off-target hits (Janas *et al.* 2018, Kobayashi *et al.* 2021, 2022, Schlegel *et al.* 2021, 2022).

Current research has shown that siRNA can bind the 3’ UTR of transcripts through a seed-mediated binding mechanism at suprapharmacological doses (Janas *et al.* 2018, Schlegel *et al.* 2021, 2022). Off-target binding of siRNA seed regions to the 3’ UTR of transcripts has been shown to lead to hepatotoxicity in rodent models and cumulative changes in the gene expression profiles that can be detected with RNA-seq (Janas *et al.* 2018, Schlegel *et al.* 2021, 2022). Despite the growing interest around developing therapeutic siRNA with limited off-target effects, there is still a lack of software specifically designed to bring together all aspects of siRNA off-target detection. For example, many algorithms have been developed to predict the binding of miRNAs to a target transcript using state-of-the-art approaches, but do not provide additional functions for visualizing or evaluating the statistical significance of those predictions in the context of a differential expression analysis (Agarwal *et al.* 2018, McGeary *et al.* 2019, Soutschek *et al.* 2022). Many popular tools, such as *TargetScan*, provide tools for miRNA target prediction and assessment, but tools are written in several languages that require an experienced bioinformatician to run (Agarwal *et al.* 2018). *SeedMatchR* is an R package developed to fill the need for a single source of accessible tools to help diagnose seed-mediated off-target effects of siRNA using RNA-seq experiments. The goal of *SeedMatchR* is to provide an easy-to-use framework for assessing siRNA off-target effects that can easily fit into new or established differential expression workflows (Supplementary Fig. S1A). *SeedMatchR* has functions to help with common bioinformatics tasks such as preparing transcriptome annotations, defining seed regions for a given siRNA, statistical testing for cumulative changes in gene expression profiles, and generating publication quality figures.

## 2 SeedMatchR workflow overview

### 2.1 Visualize siRNA and seed definitions

**Figure 1.**
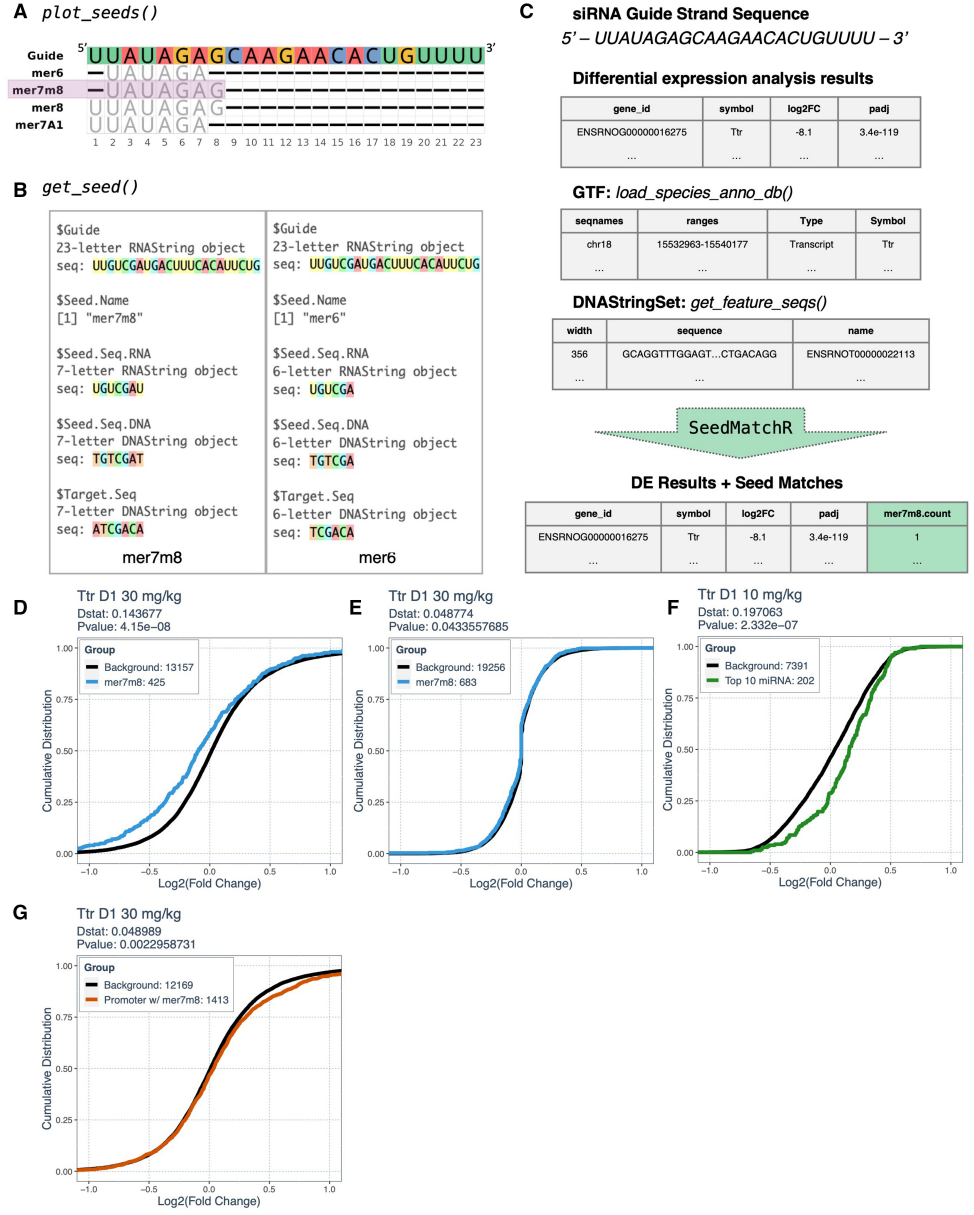
Overview of SeedMatchR functions and example applications. (A) The function *plot_seeds()* is used for exploring siRNA sequences and potential seed definitions. The purple box is highlighting the default seed (mer7m8) used by *SeedMatchR*. (B) Example outputs from *get_seed()* showing the seed sequence and target sequence that will be searched for the mer7m8 (left) and mer6 (right) seeds. (C) *SeedMatchR* inputs and outputs. There are 4 required inputs: a siRNA guide sequence, a *DESeq2* results data.frame, a species-specific GTF, and a feature specific *DNAStringSet*. The output of *SeedMatchR* is an additional column containing the seed match counts. (D) *SeedMatchR* can be used to detect siRNA seed-mediated off-target effects in RNA-seq experiments by comparing the ECDF of log_2_(fold changes) between genes with and without a seed match. A KS test is used to calculate the Dstat where the difference between the two groups is the greatest (*Dstat*: 0.138674, *P*-value: 7.74 × 10^−8^). (E) ECDF curve of log_2_(fold changes) for a siRNA glycol nucleic acid modification that helps reduce off-target effects compared to siRNA without the modification (*Dstat*: 0.049007, *P-*value: 8.239 × 10^−2^). (F) *SeedMatchR* can be used to detect cumulative changes in the expression profiles of miRNA target genes. The ECDF for miRNA targets shows a shift to the right compared to background genes (*Dstat*: 0.197063, *P*-value: 2.332 × 10^−7^). (G) *SeedMatchR* can be used to detect small activating RNA activity using promoter sequences as input. This analysis detected a positive increase in the ECDF of genes that have promoters with seed matches (*Dstat*: 0.048989, *P*-value: 2.295 × 10^−3^).

The functions available through *SeedMatchR* were designed to make it easier to explore your siRNA experiments. The *plot_seeds()* function will plot the siRNA sequence, in addition to the default seed definitions available for that siRNA (Fig. 1A). The *get_seed()* function returns an object containing the *Biostrings* sequences for a given seed definition (Fig. 1B). Alternatively, users can also define a custom seed definition by providing the start and stop position of interest. These functions are useful for reproducibly defining the target DNA sequence that is part of the string search and visualizing how it was derived (Fig. 1B).

### 2.2 Prepare required annotations

There are four required inputs for *SeedMatchR*: a *data.frame* of differential expression results, a species-specific GTF *GRanges* object, a species-specific *DNAStringSet* for genomic DNA, and a siRNA guide sequence ≥ 8 nt (Fig. 1C, Lawrence *et al.* 2013, Pages *et al.* 2023). The *SeedMatchR* package provides functions to help quickly and reproducibly generate the required annotations, taking advantage of the *Bioconductor* environment. Genomic features of interest (3’ UTR, 5’ UTR, exons, introns, or CDS) are derived from a *Txdb* object generated using the species-specific GTF file (Lawrence *et al.* 2013). Built-in *SeedMatchR* annotation databases use ENSEMBL gene IDs, but *SeedMatchR* can be used with custom annotations as well. The *SeedMatchR* documentation contains tutorials on how to generate required annotations as well as how to adjust parameters for custom gene sets.

### 2.3 Search annotations for seed matches

*Bioconductor* contains the packages *Biostrings* and *GenomicFeatures* which are tools that can be used for performing string searches in common genomic annotations (Lawrence *et al.* 2013, Pages *et al.* 2023). The function *SeedMatchR()* is designed to extend differential expression analysis workflows by annotating each gene with the number of matches found to an siRNA seed using the function *vcountpattern()* (Fig. 1A, Pages *et al.* 2023). The inputs to the primary *SeedMatchR()* function are a differential expression results *data.frame*, a GTF *GRanges* object, a *DNAStringSet* object of features to search, and the guide sequence for the siRNA. *SeedMatchR* was developed and tested on differential expression data from *DESeq2*, but any differential expression method can be used if the appropriate columns are represented.

### 2.4 Statistical analysis

The functions *de_fc_ecdf()* and *ecdf_stat_test()* are provided to streamline the comparison of empirical cumulative distribution functions (ECDF) for genes with or without a seed match (Fig. 1D). Comparison of the ECDFs for genes with a seed match to a background set has been applied to assess the effects of seed-mediated binding on gene expression profiles in miRNA and siRNA experiments (Grimson *et al.* 2007, Janas *et al.* 2018, McGeary *et al.* 2019, Schlegel *et al.* 2021, 2022). The ECDF analysis is used to gauge whether the distribution of log_2_(fold changes) observed between two sets of genes is different using the Kolmogorov–Smirnov (KS) test. Statistical testing with the KS test is performed by comparing the *D* statistic (Dstat), or difference, between the two ECDFs. In the case of seed-mediated effects, we expect the distribution of log_2_(fold changes) for transcripts with seed matches to be shifted to the left (downregulated) compared to a background set. We use a one-sided KS test to determine if the ECDF of transcripts with a seed match is less than the background distribution. The statistical functions provided can be run independently on any differential expression results looking to compare the ECDFs of different gene categories. For example, *de_fc_ecdf()* can be used to plot ECDFs for any set of genes, such as those derived from alternative binding prediction algorithms like scanMiR (Soutschek *et al.* 2022).

## 3 Example applications

### 3.1 Seed-mediated off-target analysis

*SeedMatchR* comes with functions to download and process an example dataset from siRNA experiments targeting the *Ttr* transcript in female rat liver at suprapharmacological doses (Schlegel *et al.* 2022). The purpose of this study was to demonstrate the mitigation of hepatotoxicity caused by off-target seed-binding through chemical modifications of the siRNA seed region (Schlegel *et al.* 2022). The siRNA sequence with seed driven off-target effects is named *Ttr D1*. For this analysis, *DESeq2* was used to perform differential expression analysis of rats treated with siRNA targeting the *Ttr* gene with different modifications compared to a vehicle control group (Schlegel *et al.* 2022). Each treatment condition has four biological replicates that are combined for the differential expression analysis using *DESeq2*. Using *SeedMatchR*, we can replicate the published results showing a significant shift in the ECDF for genes with a seed match compared to those without any seed matches in rats treated with 30 mg/kg of siRNA (Fig. 1D). Concordant with published results, cumulative down-regulation of predicted siRNA target genes expression was mitigated by the addition of a glycol nucleic acid at position g7 of the seed region in the siRNA called *Ttr D4* (Fig. 1E, Schlegel *et al.* 2022).

### 3.2 Predicting effects of siRNA treatment on miRNA target gene expression

siRNA and miRNA share the same AGO machinery used for binding and mediating mRNA knock-down (Gorski *et al.* 2017, Alshaer *et al.* 2021). Early studies have shown that introduction of exogenous small RNA can perturb the activity of endogenous miRNAs by co-opting the AGO2 machinery (Khan *et al.* 2009, Saito and Sætrom 2012). *SeedMatchR* can be used to assess potential off-target effects on miRNA pathways using ECDF analysis of predicted miRNA targets compared to background sets of genes (Fig. 1F). Analysis of potential perturbation of miRNA pathways requires knowledge of miRNA expression in the experimental model (Supplementary Fig. S1B). This example uses the previous dataset for the *Ttr D1* sequence as before, but we focus on the 10 mg/kg dose. We collected public data for miRNA expression in 6-week-old female rat liver available from a tissue survey of miRNA expression in rats (Yao *et al.* 2022). The read counts per miRNA were normalized by library size and averaged across replicates for the control samples in the experiment. The top 10 expressed miRNAs were considered for downstream analysis. The miRDB database was used to predict the targets of the top 10 expressed miRNAs with a target score of ≥90 (Chen and Wang 2020). Using ECDF analysis of miRNA target genes shows a positive increase in the expression of predicted miRNA targets compared to background (Fig. 1F). These finding suggest that miRNA pathways could potentially be perturbed by siRNA treatment.

### 3.3 Using SeedMatchR with any genomic feature

*SeedMatchR* functions can be used with a *DNAStringSet* for any type of feature. Since *SeedMatchR* is built on *GenomicFeatures* and *Biostrings* functions, users can use any of the built-in functions from these packages to generate custom sets of features. For example, some small RNA molecules are designed to activate gene expression through stabilizing the promoter regions of some gene bodies in the nucleus (Kwok *et al.* 2019). The main difference between these small activating RNA from siRNA is the sequence they are designed to target (Kwok *et al.* 2019). To assess the potential for gene activation, transcript features can be swapped out for genomic promoter sequences (Supplementary Fig. 1C). Users can derive promoter sequences using the function *getPromoterSeq()* from the *GenomicFeatures* package. Using the same data from previous examples for *Ttr D1* siRNA treatment at 30 mg/kg, there is a slight increase in the positive fold changes of some genes with a canonical seed match (Fig. 1G). These results suggest potential gene expression activation upon treatment of siRNA at high doses.

## 4 Conclusion

The *SeedMatchR* R package is intended to improve the reproducibility and access to commonly performed tasks in siRNA and miRNA experiments paired with RNA-seq data. The example datasets included in this publication illustrate the flexibility of *SeedMatchR* for detecting different types of off-target effects described in the literature that can attributed to different aspects siRNA-based gene knockdown.

## Supplementary Material

btae011_Supplementary_Data

## Data Availability

RNA-seq data were downloaded from the GEO accession GSE184929. The miRNA expression profiles for 6-week-old female rat liver were downloaded from GSE172269.

## References

[btae011-B1] Agarwal V , Subtelny AO, Thiru P et al Predicting microRNA targeting efficacy in drosophila. Genome Biol 2018;19:152. 30286781 10.1186/s13059-018-1504-3 PMC6172730

[btae011-B2] Alshaer W , Zureigat H, Al Karaki A et al siRNA: mechanism of action, challenges, and therapeutic approaches. Eur J Pharmacol 2021;916:174741. 10.1016/j.ejphar.2021.174178 34044011

[btae011-B3] Bartel DP. MicroRNAs: target recognition and regulatory functions. Cell 2009;136:215–33. 19167326 10.1016/j.cell.2009.01.002 PMC3794896

[btae011-B4] Chen Y , Wang X. miRDB: an online database for prediction of functional microRNA targets. Nucleic Acids Res 2020;48:D127–d131. 31504780 10.1093/nar/gkz757 PMC6943051

[btae011-B5] de Brito E , Cunha D, Frederico ABT, Azamor T, Frederico JG et al Biotechnological evolution of siRNA molecules: from bench tool to the refined drug. Pharmaceuticals (Basel) 2022;15. 10.3390/ph15050575 PMC9146980 35631401

[btae011-B6] Gorski SA , Vogel J, Doudna JA. RNA-based recognition and targeting: sowing the seeds of specificity. Nat Rev Mol Cell Biol 2017;18:215–28. 28196981 10.1038/nrm.2016.174

[btae011-B7] Grimson A , Farh KK-H, Johnston WK et al MicroRNA targeting specificity in mammals: determinants beyond seed pairing. Mol Cell 2007;27:91–105. 17612493 10.1016/j.molcel.2007.06.017 PMC3800283

[btae011-B8] Hu B , Zhong L, Weng Y et al Therapeutic siRNA: state of the art. Signal Transduct Target Ther 2020;5:101. 32561705 10.1038/s41392-020-0207-x PMC7305320

[btae011-B9] Janas MM , Schlegel MK, Harbison CE et al Selection of GalNAc-conjugated siRNAs with limited off-target-driven rat hepatotoxicity. Nat Commun 2018;9:723. 29459660 10.1038/s41467-018-02989-4 PMC5818625

[btae011-B10] Khan AA , Betel D, Miller ML et al Transfection of small RNAs globally perturbs gene regulation by endogenous microRNAs. Nat Biotechnol 2009;27:549–55. 19465925 10.1038/nbt.1543 PMC2782465

[btae011-B11] Kobayashi Y et al Selection of chemical modifications in the siRNA seed region that repress off-target effect. Methods Mol Biol 2021;2282:17–30. 33928567 10.1007/978-1-0716-1298-9_2

[btae011-B12] Kobayashi Y , Tian S, Ui-Tei K. The sirna off-target effect is determined by base-pairing stabilities of two different regions with opposite effects. Genes 2022;13:319. 35205363 10.3390/genes13020319 PMC8872465

[btae011-B13] Kwok A , Raulf N, Habib N. Developing small activating RNA as a therapeutic: current challenges and promises. Ther Deliv 2019;10:151–64. 30909853 10.4155/tde-2018-0061

[btae011-B14] Lawrence M , Huber W, Pagès H et al Software for computing and annotating genomic ranges. PLoS Comput Biol 2013;9:e1003118. 23950696 10.1371/journal.pcbi.1003118 PMC3738458

[btae011-B15] McGeary SE , Lin KS, Shi CY et al The biochemical basis of microRNA targeting efficacy. Science 2019;366:eaav1741. 10.1126/science.aav1741 PMC7051167 31806698

[btae011-B16] Pages HAP , Gentlemen R, DebRoy S. Biostrings: efficient manipulation of biological strings. Release 2023;2.

[btae011-B17] Saito T , Sætrom P. Target gene expression levels and competition between transfected and endogenous microRNAs are strong confounding factors in microRNA high-throughput experiments. Silence 2012;3:3. 22325809 10.1186/1758-907X-3-3 PMC3293725

[btae011-B18] Schlegel MK , Janas MM, Jiang Y et al From bench to bedside: improving the clinical safety of GalNAc-siRNA conjugates using seed-pairing destabilization. Nucleic Acids Res 2022;50:6656–70. 35736224 10.1093/nar/gkac539 PMC9262600

[btae011-B19] Schlegel MK , Matsuda S, Brown CR et al Overcoming GNA/RNA base-pairing limitations using isonucleotides improves the pharmacodynamic activity of ESC+ GalNAc-siRNAs. Nucleic Acids Res 2021;49:10851–67. 34648028 10.1093/nar/gkab916 PMC8565336

[btae011-B20] Soutschek M , Gross F, Schratt G et al scanMiR: a biochemically based toolkit for versatile and efficient microRNA target prediction. Bioinformatics 2022;38:2466–73. 35188178 10.1093/bioinformatics/btac110

[btae011-B21] Yao X , Sun S, Zi Y et al Comprehensive microRNA-seq transcriptomic profiling across 11 organs, 4 ages, and 2 sexes of Fischer 344 rats. Sci Data 2022;9:201. 35551205 10.1038/s41597-022-01285-7 PMC9098487

