# Supplementary figures and images for: SeedMatchR: identify off-target effects mediated by siRNA seed regions in RNA-seq experiments
Source: Bioinformatics. 2024 Jan 8;40(1):btae011. doi: 10.1093/bioinformatics/btae011 (PMC10799297; doi:10.1093/bioinformatics/btae011)

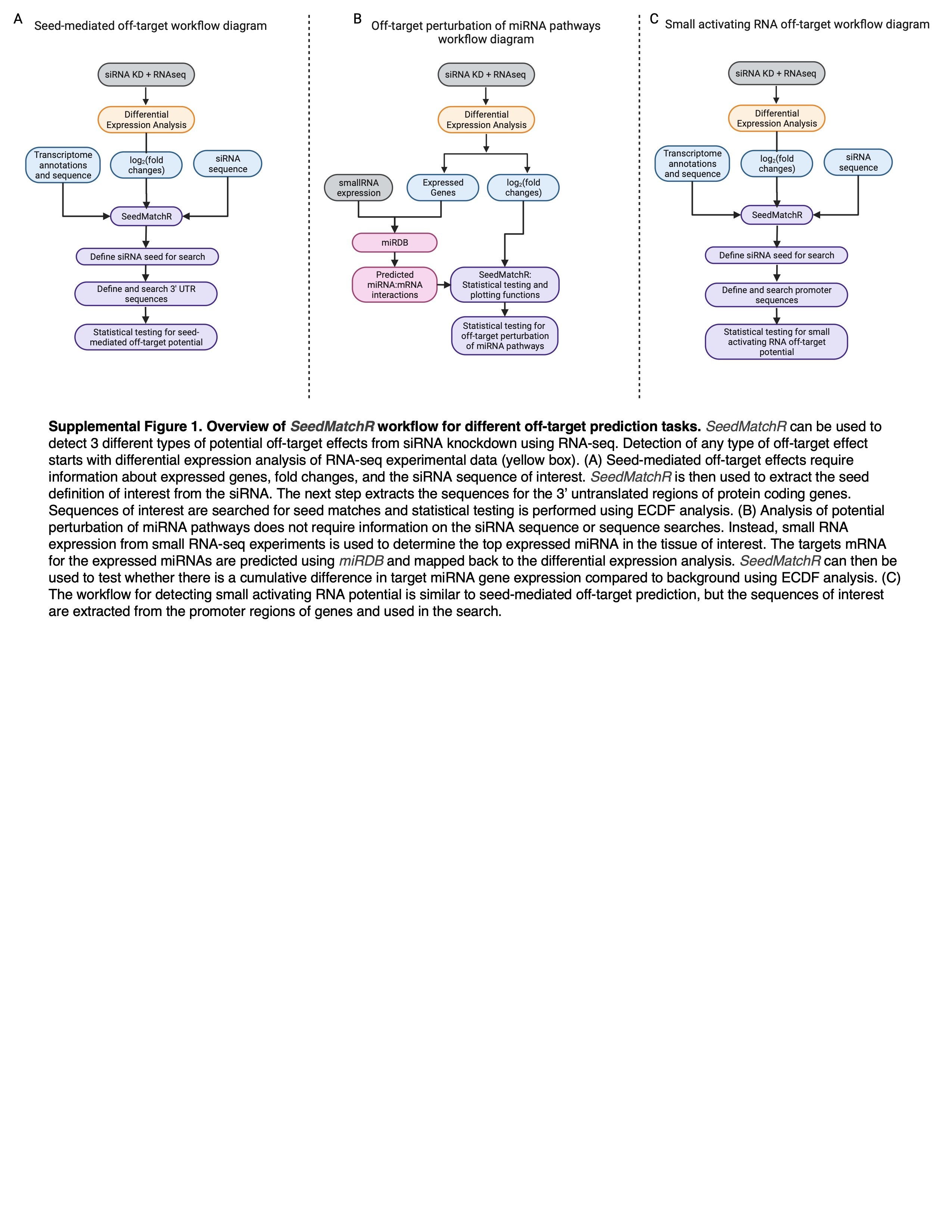

Supplement: btae011_Supplementary_Data [file btae011_supplementary_data.zip › SeedMatchR_SI1.jpg]
